# Attractant potential of *Enterobacter cloacae* and its metabolites to *Bactrocera dorsalis* (Hendel)

**DOI:** 10.3389/fphys.2024.1465946

**Published:** 2024-10-03

**Authors:** Yawen Duan, Anjuan Li, Lin Zhang, Chongwen Yin, Zhihong Li, Lijun Liu

**Affiliations:** ^1^ College of Plant Protection, China Agricultural University, Beijing, China; ^2^ Key Laboratory of Surveillance and Management for Plant Quarantine Pests, Ministry of Agriculture and Rural Affairs, Beijing, China; ^3^ Institute of Sanya, China Agricultural University, Sanya, China

**Keywords:** oriental fruit fly, gut microorganism, *Enterobacter cloacae*, attractant effect, L-prolinamide, synergistic effect

## Abstract

**Objective:**

*Bactrocera dorsalis* (Hendel) has a wide host range. It has been the most important quarantine pest in many countries or regions. Currently, chemical control and bait trapping are mainly used in the monitoring, prevention, and control of *B. dorsalis*. However, chemical control will cause pollution of the environment and drug resistance of insects. Methyl eugenol, the main attractant currently used, can only attract males of *B. dorsalis*.

**Methods:**

This study focused on the attractant function and active substances of one key intestinal bacterium, *Enterobacter cloacae*, which was isolated from *B. dorsalis*.

**Results:**

First, the attraction of the *E. cloacae* autoclaved supernatant to male and female adults of 0, 6, and 15 days post-emergence was confirmed using a Y-type olfactometer. Subsequently, through metabolome sequencing and bioassays, L-prolinamide was identified and confirmed as the most effective attractant for *B. dorsalis*. Finally, the synergistic effect of L-prolinamide with the sex attractant ME was validated through field experiments. This study confirmed the attraction effect of *E. cloacae* on *B. dorsalis* and also proved the attraction effect of L-prolinamide, the metabolite of *E. cloacae*, on *B. dorsalis*. This laid a theoretical foundation for the development of a new attractant and safe, green, and efficient prevention and control technology of *B. dorsalis*.

## 1 Introduction

*Bactrocera dorsalis* (Hendel) belongs to the Diptera (Tephritidae) family. Due to the direct damage it causes to fruits and vegetables and its negative effect on imports and exports, *B. dorsalis* has become one of the world’s most destructive agricultural pests and a major impediment to international fresh commodity trade (Qin et al., 2018). It has caused massive economic losses to agricultural production in tropical and subtropical countries or regions. *Bactrocera dorsalis* can damage over 40 families and 250 genera of fruits and vegetables due to its extensive host range (Liu et al., 2017). It causes fruit rot and premature fruit drop due to female adults laying eggs in the fruits and larvae eating the fruits before pupating (Qin et al., 2018; Verghese et al., 2012; Chen and Ye, 2007). Therefore, the prevention and management of this notorious pest are of great significance. At present, chemical, biological, and agricultural methods are employed, with chemical control prevailing due to its higher efficiency. Trapping males using methyl eugenol (ME), the main sexual attractant used currently, is also an effective method for population control. However, chemical control can also cause pollution to the environment and induce a high level of drug resistance in insects. Moreover, attractants can only attract males. Therefore, it is necessary to explore new environmentally friendly control methods and new efficient attractants.

Many microbes play a critical role in the growth and evolution of insects (Genta et al., 2006; Wang et al., 2011). Many studies have shown that four major phyla of intestinal bacteria, namely, Bacteroidetes, Actinobacteria, Firmicutes, and Proteobacteria, were found in the intestine of *B. dorsalis* (Hadapad et al., 2019; Colman et al., 2012; Liu et al., 2016). Several gut microbes have an attractive effect on fruit flies and play an essential role in the chemical exchange between individuals. Wang et al. (2014) have identified the intestinal bacteria of *B. dorsalis* adults by the PCR-DGGE method and confirmed the attraction effect of these bacteria on *B. dorsalis*. Their results indicated that Enterobacteriaceae, Enterococcaceae, and Bacillariophyceae had attraction effects on *B. dorsalis*, among which *Enterobacter cloacae* had a better attraction for *B. dorsalis*. It has been reported that the volatile substance 2-butanone produced by *Klebsiella pneumoniae*, *Citrobacter fumigatus*, and *E. cloacae* can attract *B. dorsalis* (Jang and Nishijima, 1990). It has also been found that metabolites of *K. pneumoniae*, *K. oxytoca*, and *Raoultella terrigena* isolated from the reproductive system of female *B. dorsalis* had an attraction effect on *B. dorsalis*, but the active substance responsible has not yet been confirmed (Shi et al., 2012; Shi et al., 2012).

Using attractants to control and prevent fruit flies can reduce the use of chemicals and, hence, alleviate the problems caused by pesticide resistance. Based on different attracting targets, attractants include male, female, and bisexual attractants. Most of the current male attractants are plant-derived volatiles or paraphylactones, which are usually used to attract males in the field or to monitor populations (Vargas et al., 2010), among which ME, ME analogs, Capilure, trimedlure, Zingerone [4-(4-hydroxy-3-methoxyphenyl)-2-butanone], and cuelure (CL) are the main known species (Zhang, 1991; Park et al., 2020). Until now, ME can be obtained from more than 450 kinds of plant releases, and it has been the most widely used *B. dorsalis* sex attractant (Tan et al., 2011). More than 80 species of fruit fly males, including *B. carambolae*, *B. correcta*, *B. invadens*, and *B. dorsalis*, are strongly attracted to ME (Tan et al., 2011; Shelly, 2010). A study has shown that the ME ingested by males can be converted into several metabolites, which are stored in their glands and act as components of the male sex pheromone (Nishida et al., 1988). In addition, ME plays an important role in enhancing the mating ability and total protein content of *B. dorsalis* males (Ji et al., 2013; Reyes-Hernández et al., 2019). Intake of ME by *B. carambolae* males will strengthen the sexual selection of poplar fruit flies (Wee et al., 2007). CL was initially noted to have a significant attraction effect on *Zeugodacus cucurbitae*, and the subsequent test revealed that raspberry ketone was not as effective as CL (Beroza et al., 1960; Keiser et al., 1973; Drew, 2010). However, ME and CL poorly attract some fruit fly males, such as *B. xanthodes* (Broun) and *B. halfordiae* (Royer et al., 2019a; Royer et al., 2019b). It was also demonstrated that some eugenol analogs (isoeugenol, ME, and dihydroeugenol) and zinc ketone were more effective male attractants than CL or ME for some fruit flies, with ME showing an effective attraction more than 50 times higher than ME in attracting *Bactrocera xanthodes* (Royer et al., 2019b; Royer et al., 2018). The United States Department of Agriculture has developed trimedlure (TML), which has been widely used as an attractant for *Ceratitis capitata*. TML is an isomeric mixture of 4-(and 5-) chloro-2-methylcyclo-hexane-1-carboxylate. Among them, (1*R*,2*R*,4*S*) tert-butyl 4-chloro-2-methylcyclohexane-1-carboxylate was considered to be the most effective attractant (Jang et al., 2005), and its analog ceralure has also been found to be effective in attracting *C. capitata*. However, due to the difficulty of its synthesis, the most widely used attractant for Mediterranean fruit fly is still TML (Jang et al., 2005). However, most of these attractants are male attractants and can only attract males. There are also some female or bisexual attractants that can attract females or both males and females. These attractants are usually intraspecific pheromones, host volatiles (Fein et al., 1982), food source attractants (Heath et al., 1995), associated bacterial ferments, and secondary metabolites (Drew et al., 1983). Most of the reported intraspecific pheromones with female attraction potential were sex pheromones, which were obtained by extracting rectal gland isolates and were usually composed of pheromones released by male adults (Zhang et al., 2019). The role of female fruit flies is critical for population growth. Hence, despite the effectiveness of ME in attracting male fruit flies, a need exists for attractants that can target both sexes and reduce the environmental impact. *Enterobacter cloacae* and its metabolites may offer advantages in the attractant market by providing a stronger and longer-lasting attractant effect with lower environmental impact and production costs. Additionally, the natural sources and unique mechanisms of action of these metabolites could make them an effective and sustainable alternative. Thus, research on female or bisexual attractants is very important for the development of new green environment-friendly control technology of fruit flies.

In this study, a laboratory-reared population was utilized to investigate the attraction effect of *E. cloacae*, a key intestinal bacterium isolated from *B. dorsalis*, on host fruit flies at various developmental stages. Active substances with attraction properties were screened through metabolome analysis of the supernatant from *E. cloacae*. Synergistic effect of the active substances on ME was also investigated through a field experiment. This study reported for the first time that L-prolinamide has an attraction effect on *B. dorsalis*, and it has a synergistic effect on the sexual attractant named ME, which is important for further development of green and efficient attractants.

## 2 Materials and methods

### 2.1 Insects

Adults of *B. dorsalis* were initially collected from Guangzhou, Guangdong province, and the populations have been reared in the laboratory for more than 15 generations. Larvae were fed with artificial diets following the description of Bai et al. (2019). The adults were fed with a powdered diet (peptone: sucrose = 1: 3) after emergence and provided with deionized water using moist cotton balls. The larvae (4-day-old) and adult males and females (0, 6, and 15 days post-emergence) of laboratory-reared *B. dorsalis* were collected for this experiment.

### 2.2 Bacteria

*Bactrocera dorsalis* adults at 10 days post-eclosion were selected and washed with sterile enzyme-free water to clean their body surfaces. Sequentially, surface disinfection was performed using the 1% sodium hypochlorite solution and 75% ethanol solution, each for 1 min, followed by three washes with enzyme-free sterile water to remove residual solutions. The flies were then placed on ice for storage. In a sterile Petri dish, PBS buffer solution was added, and the midgut was dissected using dissecting forceps. The gut and remaining tissues were placed in separate sterile centrifuge tubes, with five guts combined as one biological replicate. After grinding in liquid nitrogen, PBS was added to create a grinding solution. Subsequently, 100 μL of the gut-grinding solution was pipetted onto NA culture medium and spread evenly. Following incubation at 37°C for 2 days, single bacterial colonies on the plates were purified. After two rounds of purification, single colonies were selected and cultured in the NB liquid medium. Bacterial DNA was extracted using a bacterial genome DNA extraction kit (Tiangen, Beijing, China), amplified using universal bacterial 16S rDNA primers (Table 1), and aligned with NCBI sequences. Physiological and biochemical identification kits specific to Enterobacteriaceae (SHBG08, Qingdao Hope Bio-Technology Co., Ltd., China) confirmed the strain as *E. cloacae*. *Enterobacter cloacae* was mixed with glycerin and stored at −80°C until reused. The strain can be revived using LB liquid medium before being used. The bacterial solution was obtained after being cultured for 48 h under the culture condition of 37°C and 220 r/min.

**TABLE 1:** 16S common primers.

Name	Sequence
27F	AGA​GTT​TGA​TCC​TGG​CTC​AG
1492R	GGT​TAC​CTT​GTT​ACG​ACT​T

### 2.3 Attracting ability of *E. cloacae* on larvae of *B. dorsalis*

**FIGURE 1:**
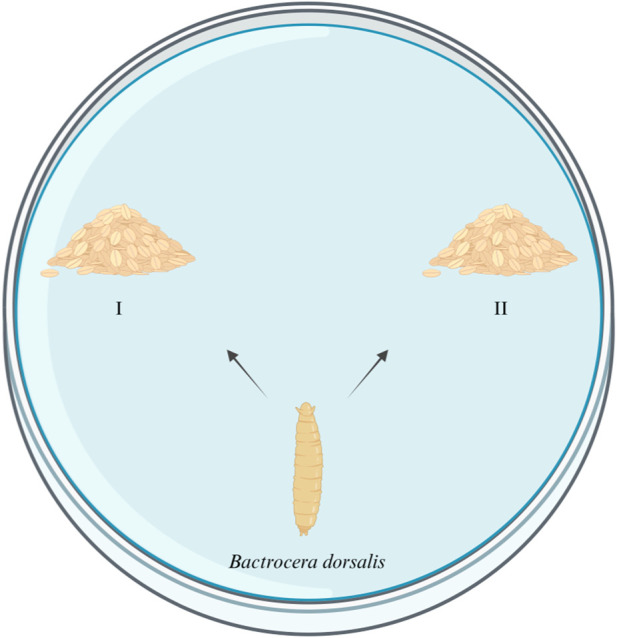
Attraction test of *E. cloacae* on the larvae of *B. dorsalis*. I: experimental group. II: control group.

Four kinds of artificial food were used for this experiment, including food which has been eaten by the same group of larvae (A), food which has been eaten by another group of larvae (B), fresh food with the *E. cloacae* supernatant added (C), and fresh food with nothing added as control (D). The formula and the condition of the food are listed in Table 2. A, B, and C are the experimental groups, and D is the control group. Five grams of food from the experiment group (A, B, or C) and control group (D) were put in the two sides of a 9-cm culture dish, respectively. Five 4-day-old larvae were placed at the mid-point between the two kinds of food each time, while their feeding choices were recorded. The condition that the larvae arrive at one group and feed, without secondary selection, can be recorded as a valid selection. Ten replicates were set up for each group (Figure 1).

**TABLE 2:** Samples of attractor test on larvae.

Ingredient	A	B	C	D
Sucrose (g)	62.5	62.5	62.5	62.5
Brewer’s yeast powder(g)	15.5	15.5	15.5	15.5
Wheat bran(g)	117.5	117.5	117.5	117.5
Sorbic acid(g)	0.5	0.5	0.5	0.5
Ascorbic acid(g)	0.25	0.25	0.25	0.25
Methylparaben(g)	0.3	0.3	0.3	0.3
Double-distilled water (mL)	300	300	300	—
*E. cloacae* supernatant (mL)	—	—	—	300
Fed by the larvae (themselves)	Yes	No	No	No
Fed by the larvae (other than themselves)	No	Yes	No	No

### 2.4 Attracting ability of *E. cloacae* on adults of *B. dorsalis* with different developing periods

**FIGURE 2:**
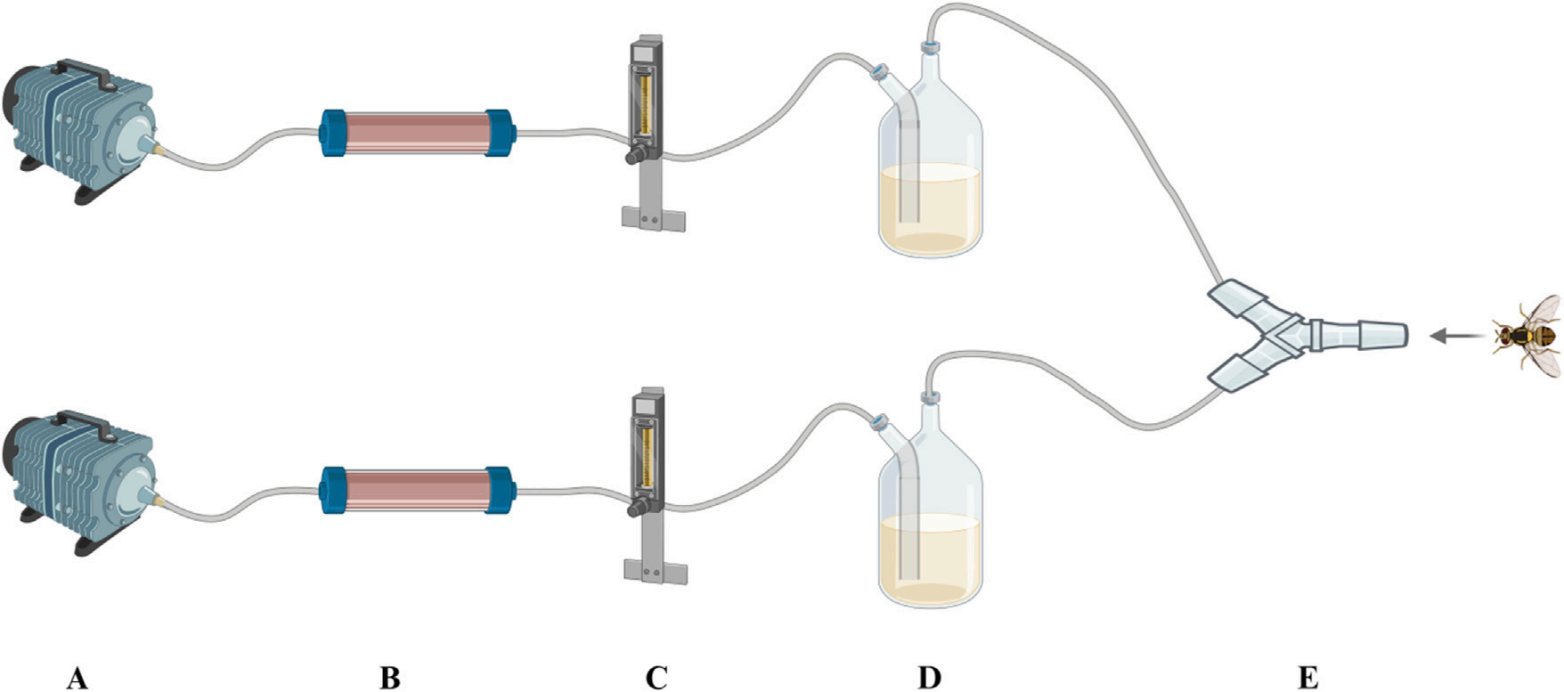
Self-made Y-type olfactometer. **(A)** Air pump, **(B)** active carbon filter, **(C)** gas flowmeter, **(D)** reagent bottle, and **(E)** Y-tube.

Two kinds of attractant and double-distilled water were used for this test, including the *E. cloacae* supernatant (A), autoclave sterilized *E. cloacae* supernatant (B), and double-distilled water as control (C). The detailed composition of these attractants are listed in Table 3. A self-made Y-type olfactometer was used to test the attraction effect on male and female *B. dorsalis* at 0, 6, and 15 days post-emergence. The self-made Y-type olfactory instrument is composed of an air pump, a pressure regulator tank (Outstanding, China), a gas flowmeter (Shuanghuan, China), an activated carbon tube, a sample bottle, and a Y-type tube (Crystal Technical, China). The Y-type tube has a handle length of 20 cm, an arm length of 10 cm, and an angle of 60°. The two arms of the Y-type tube are equipped with fluorescent lamps. It was ensured that both arms have the same light intensity. The protocol is shown in Figure 2, and the airflow rate was maintained at 100 mL/min. The condition that the adult entered the Y-tube for more than 1 cm and stayed for more than 15 s was recorded as a valid selection. After 30 valid selections in each group, the device was disassembled and rinsed with anhydrous ethanol and 75% alcohol and dried. Then, the sample bottles were switched, and the test was repeated. There were three replicates recorded for each developing period and sex. This part of the study involved the use of larvae (4-day-old) and adult males and females (0, 6, and 15 days post-emergence) of *B. dorsalis*. Each group had 30 valid samples.

**TABLE 3:** Samples of attractor test on adults.

Component	A	B	C
*E. cloacae* supernatant (mL)	50	—	—
Autoclaved supernatant of *E. cloacae* (mL)	—	50	—
Double-distilled water (mL)	—	—	50

### 2.5 Preparation of metabolome samples of *E. cloacae*

The bacteria solution, after being cultured for 48 h (37°C, 220 r/min) using LB liquid media, was centrifuged at 10,000 r/min for 15 min, and 200 mL of the supernatant was used as sample 1. An additional 200 mL of the supernatant was autoclaved at 121°C for 20 min and used as sample 2. There were six replicates for each sample. Finally, the processed samples were used for the LC–MS non-targeted metabolomics study (Shanghai Majorbio Bio-Pharm Technology Co., Ltd.). A measure of 25 mg of solid sample was added to a 2-mL centrifuge tube, and a 6-mm-diameter grinding bead was added. An amount of 400 μL of the extraction solution [methanol: water = 4:1 (v:v)] containing 0.02 mg/mL of the internal standard (L-2-chlorophenylalanine) was used for metabolite extraction. Samples were ground by the Wonbio-96c (Shanghai Wanbo biotechnology co., Ltd.) frozen tissue grinder for 6 min (−10°C, 50 Hz), followed by low-temperature ultrasonic extraction for 30 min (5°C, 40 kHz). The samples were left at −20°C for 30 min, centrifuged for 15 min (4°C, 13,000 g), and the supernatant was transferred to the injection vial for LC–MS/MS analysis. An amount of 20 μL of the supernatant was removed from each sample and mixed as a quality control sample (QC) with the same volume as the sample. The QC samples were disposed and tested in the same manner as the analytic samples. It helped represent the whole sample set, which would be injected at regular intervals (every 5–15 samples) in order to monitor the stability of the analysis.

### 2.6 Analysis of the metabolome of *Enterobacter cloacae*

#### 2.6.1 Liquid chromatography (LC)–mass spectrometry (MS)/MS analysis

The LC–MS/MS analysis of the sample was conducted on a Thermo UHPLC-Q Exactive HF-X system equipped with an ACQUITY HSS T3 column (100 mm × 2.1 mm i.d., 1.8 μm; Waters, United States) at Majorbio Bio-Pharm Technology Co. Ltd. (Shanghai, China). The mobile phases consisted of 0.1% formic acid in water: acetonitrile (95:5, v/v) (solvent A) and 0.1% formic acid in acetonitrile: isopropanol: water (47.5:47.5, v/v) (solvent B). The flow rate was 0.40 mL/min, and the column temperature was 40°C. The injection volume was 3 μL.

The mass spectrometric data were collected using a Thermo UHPLC-Q Exactive HF-X Mass Spectrometer equipped with an electrospray ionization (ESI) source operating in the positive mode and negative mode. The optimal conditions were set as follows: aux gas heating temperature at 425°C; capillary temp at 325°C; sheath gas flow rate at 50 psi; aux gas flow rate at 13 psi; ion-spray voltage floating (ISVF) at −3,500 V in negative mode and 3,500 V in positive mode; normalized collision energy, 20–40–60 eV rolling for MS/MS. The full MS resolution was 60,000, and the MS/MS resolution was 7,500. Data acquisition was performed in the data-dependent acquisition (DDA) mode. The detection was carried out over a mass range of 70–1,050 m/z.

#### 2.6.2 Substance identification and analysis

The UHPLC-MS raw data were converted into the common format by Progenesis QI software (Waters, Milford, United States) through baseline filtering, peak identification, peak integral, retention time correction, and peak alignment. Then, the data matrix containing sample names, m/z, retention time, and peak intensities was exported for further analyses. At the same time, the metabolites were identified by searching the databases, and the main databases were the HMDB (http://www.hmdb.ca/), Metlin (https://metlin.scripps.edu/), and the self-compiled Majorbio Database (MJDB) of Majorbio Biotechnology Co., Ltd. (Shanghai, China).

The data matrix obtained by searching databases was uploaded to the Majorbio cloud platform (https://cloud.majorbio.com) for data analysis. First, the data matrix was pre-processed as follows: at least 80% of the metabolic features detected in any set of samples was retained. After filtering, the minimum value in the data matrix was selected to fill the missing value, and each metabolic signature was normalized to the sum. To reduce the errors caused by sample preparation and instrument instability, the response intensities of the sample mass spectrometry peaks were normalized using the sum normalization method to obtain the normalized data matrix. Meanwhile, the variables of QC samples with relative standard deviation (RSD) > 30% were excluded and log10 logarithmicized to obtain the final data matrix for subsequent analysis. Then, through R software ropls tools, the data matrix of input samples was used for PCA analysis, which generally reflected the metabolic differences among samples in each group and the variation degree among samples in the group (Cala et al., 2018). OPLS-DA analysis was used to eliminate noise information that is not relevant to classification and also obtain relevant metabolite information that causes significant differences between the two groups (Cala et al., 2018). To visualize the content of metabolites, metabolites were clustered according to their content in each sample. At the same time, metabolites were classified according to their participation in the pathway or the function they perform using the KEGG database. The metabolically concentrated metabolites are displayed on the KEGG pathway map (Li et al., 2018).

### 2.7 Field experiment

The field experiment was conducted in Yazhou, Sanya City, Hainan Province, from October to November of 2023. A total of eight traps, where four traps were the control group and another four traps were the treatment group, were randomly hung on one of the branches in the sunshade side of the green canopy. The hanging height was between 1 m and 1.5 m from the ground. For control groups, only the absorbent cotton, in which 3 mL ME was added, was placed in the small tank. However, for the treatment group, in addition to absorbent cotton with 3 mL ME, the bottom was filled with 100 mL L-prolinamide solution with a final concentration of 3%. The traps were hung on the same branch, and the distance between the control and treatment group was approximately 0.5 m. The numbers, species, and the sex ratio of flies were investigated every 7 days, and the traps were changed. There were four biological replications for this experiment, and the experiment was repeated four times.

### 2.8 Statistical analysis

SPSS statistical analysis software was used to analyze the relevant experimental data using nonparametric tests. S was used as the selection rate, and the calculation method is explained herein. The term “average number of *B. dorsalis* attracted” refers to the number of adults who enter the Y-tube side for more than 1 cm and stay for more than 15 s. The term “total number of *B. dorsalis*” refers to the 30 valid selections of the adult in each group.

$$S\left(\%\right)=\frac{\text{Average}\;\text{number}\;\text{of}\;B.\;dorsalis\;\text{attracted}\;\text{to}\;\text{the}\;\text{treatment}\;\text{group}\;\left(\text{or}\;\text{control}\;\text{group}\right)}{\text{Total}\;\text{number}\;\text{of}\;B.\;dorsalis}\times 100.$$

## 3 Results

### 3.1 Response of 4-day-old *B. dorsalis* larvae to various types of artificial food

**FIGURE 3:**
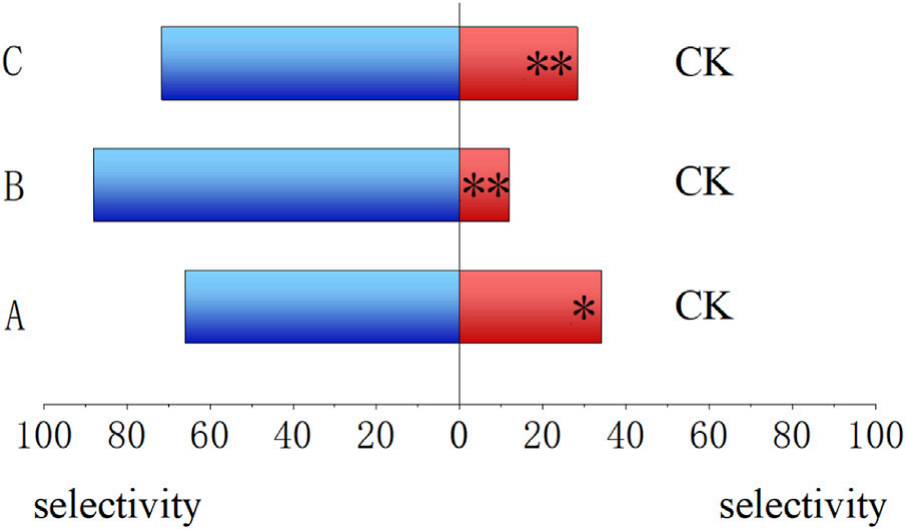
Feeding trend of *Bactrocera dorsalis* larvae for different larval feeds. **(A)** Food that has been fed by themselves for 4 days. **(B)** Food that has been fed by the larvae other than themselves for 4 days. **(C)** Fresh food prepared using the *Enterobacter cloacae* solution. (CK) Fresh food prepared using ddH_2_O. *: *P* < 0.05; **: *P* < 0.01.

As shown in Figure 3, compared to CK, the 4-day-old larvae preferred to feed on treated artificial food. The food that has been fed by other insects for 4 days showed the highest selectivity (88.00%, *P* < 0.01), followed by the fresh food prepared with *E. cloacae* solution (72%, *P* < 0.01) and the food that has been fed by themselves for 4 days (66.00%, *p* = 0.015 < 0.05).

### 3.2 Attraction effect of *E. cloacae* on adult *B. dorsalis*

**FIGURE 4:**
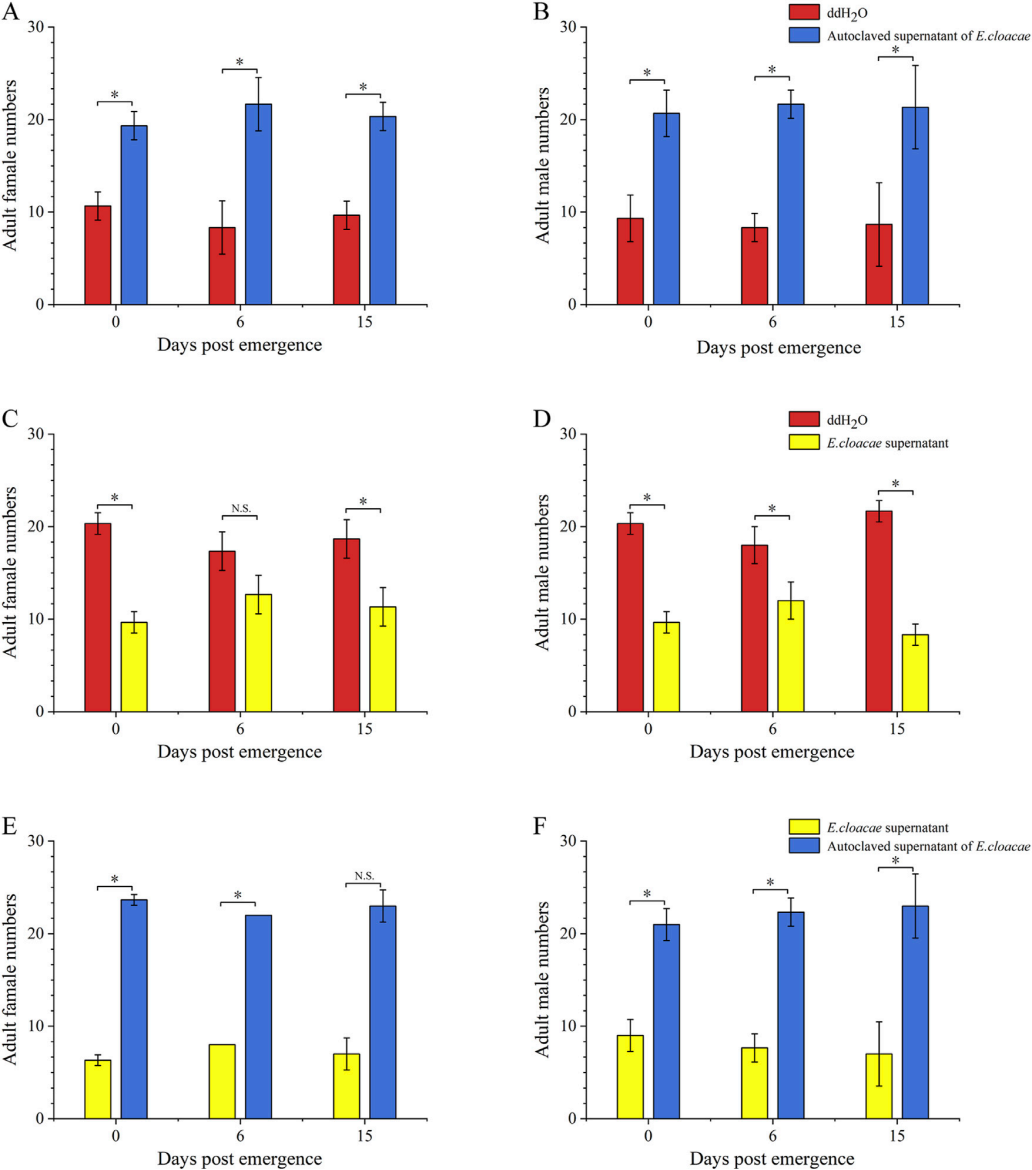
Attractant of *Enterobacter cloacae* to adults of *Bactrocera dorsalis*. Red: ddH_2_O; yellow: supernatant of *Enterobacter cloacae*; blue: autoclaved supernatant of *E. cloacae.* **(A)**: attraction of Enterobacter cloacae supernatant which has been autoclave sterilized to female *B. dorsalis*. **(B)**: attraction of *E. cloacae* supernatant which has been autoclave sterilized to male *B. dorsalis*. **(C)**: attraction of *E. cloacae* supernatant to female *B. dorsalis*. **(D)**: attraction of *E. cloacae* supernatant male *B. dorsalis*. **(E)**: attraction of *E. cloacae* supernatant which has been autoclave sterilized and *E. cloacae* supernatant to female *B. dorsalis*. **(F)**: attraction of *E. cloacae* supernatant which has been autoclave sterilized and *E. cloacae* supernatant to male of *B. dorsalis*. *: *P* < 0.05; **: *P* < 0.01.

Compared to double-distilled water and the *E. cloacae* supernatant, the autoclaved supernatant of *E. cloacae* showed a significantly increased attraction ability for adults of *B. dorsalis* at 0, 6, and 15 days post-emergence, including females (Figures 4A, E) and males (Figures 4B, F). Additionally, the number of *B. dorsalis* attracted by double-distilled water was significantly higher than that attracted by the supernatant of bacteria, except for 6-day-old females (Figures 4C, D).

### 3.3 Comparative metabolome analysis of the supernatant and autoclaved supernatant of *E. cloacae*

#### 3.3.1 PCA and orthogonal partial least-squares discrimination (OPLS-DA) analysis

**FIGURE 5:**
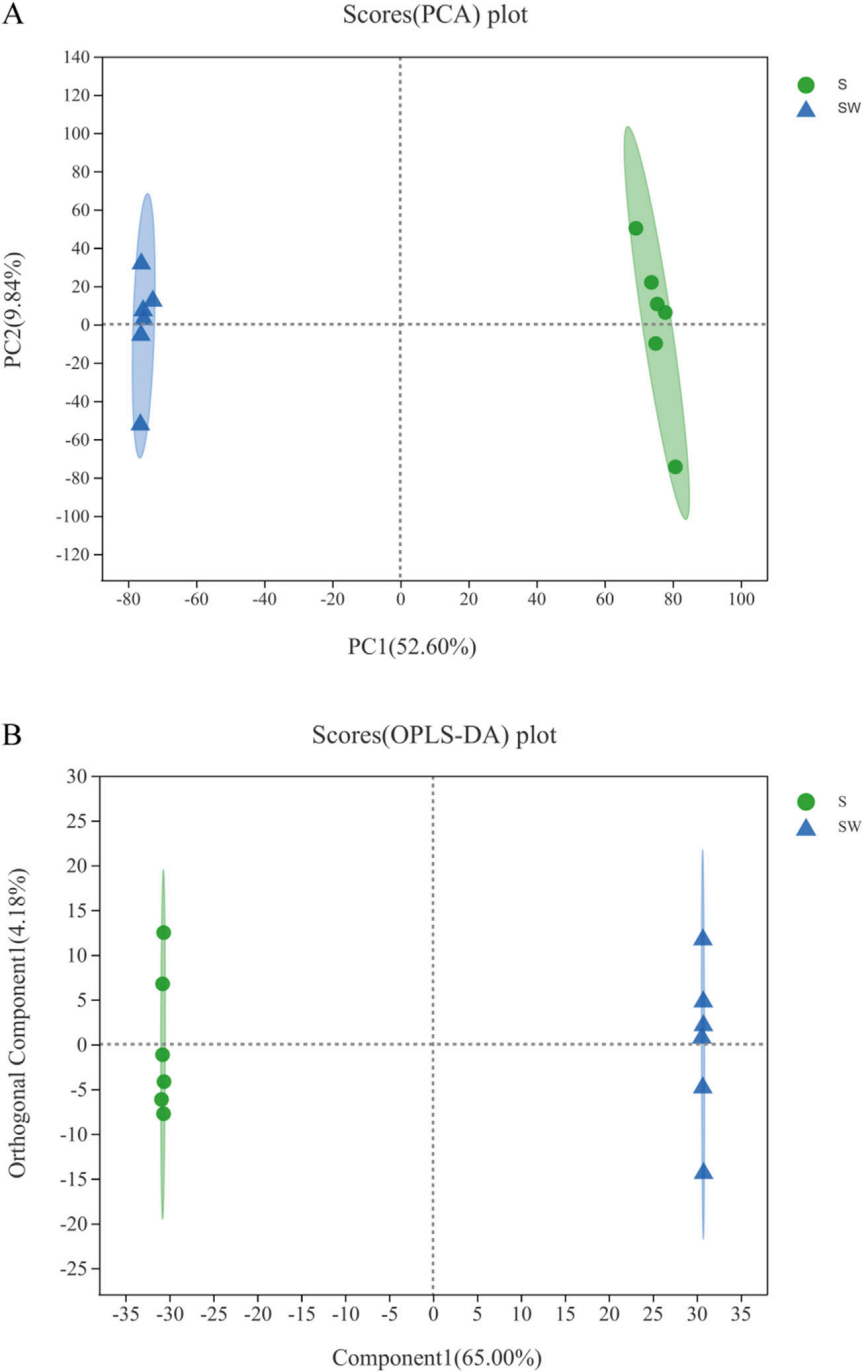
Scatter points of PCA **(A)**. Dispersion points of OPLS-DA **(B)**. (S) Experimental group: *Enterobacter cloaca* supernatant that has been autoclave sterilized. (SW) Control group: *Enterobacter cloaca* supernatant. The confidence ellipse represents the group of “true” samples with 95% confidence, and the samples beyond this region can be considered as possible abnormal samples.

As shown in Supplementary Figure S1, the total ion overlap chromatograms of the quality control samples in the positive and negative ion modes had good peak shapes and relatively uniform distribution, which represent a high quality of the metabolome. The PCA analysis allows us to observe the separation trend between groups and the presence of outliers in the experimental model and reflects the inter- and intra-group variability from the raw data. The treated group was farther apart from the control, and the biological repetitions in the treated or control group are closer together (Figure 5A).

The OPLS-DA method can distinguish the test group from the control group (R2X = 0.65, R2Y = 1, Q2 = 0.995) with good stability, as shown in Figure 5B. All samples in the test and control groups were within the 95% confidence interval, indicating that the accuracy of these data is relatively high. Both PCA and OPLS-DA analysis showed that the differences between the test and control groups are more pronounced and the intra-group replicates are more stable.

#### 3.3.2 Heatmap analysis of differential metabolites

**FIGURE 6:**
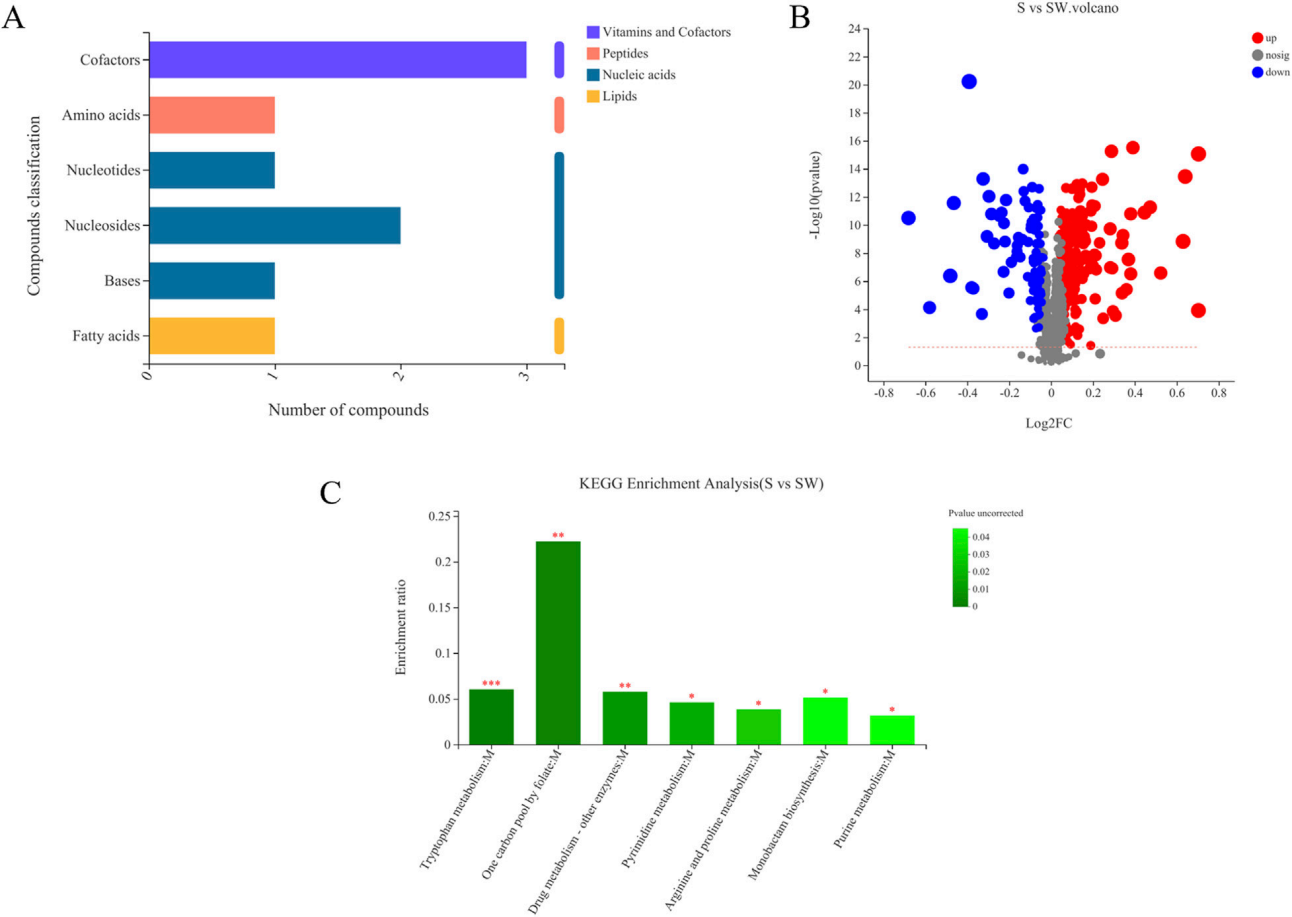
Volcanic map of differential metabolites in different samples **(A)**. (S) Experimental group (*Enterobacter* cloaca supernatant that has been autoclave sterilized). (SW) Control group (*E. cloaca* supernatant). Classification of KEGG compounds **(B)**. The ordinate is the secondary classification category of KEGG compounds. The horizontal coordinate is the number of metabolites annotated to this classification. KEGG pathway enrichment of differential metabolites in experimental group and metabolite **(C)**. The abscissa represents the pathway name, and the ordinate represents the enrichment rate, indicating the ratio of the number of metabolites enriched in this pathway to the number of metabolites annotated into the pathway. The greater the ratio, the greater the degree of enrichment. The color gradient of the column represents the significance of enrichment, and the darker the color, the more significant the enrichment of the KEGG term. *P* value < 0.001 is marked with ***, *P* value < 0.01 is marked with **, and *P* value < 0.05 is marked with *.

Differential metabolites between the autoclaved supernatant and supernatant of *E. cloacae* were screened based on univariate statistical analysis and VIP values. A total of 302 differential metabolites were screened (*P* < 0.05 and VIP values > 1), of which 208 were upregulated (VIP > 1 and fold change > 1) and 94 were downregulated (VIP > 1 and 0 < fold change < 1) (Figure 6A). Among them, FC > 1 and the metabolites with clear species are provided in Supplementary Table S1.

**FIGURE 7:**
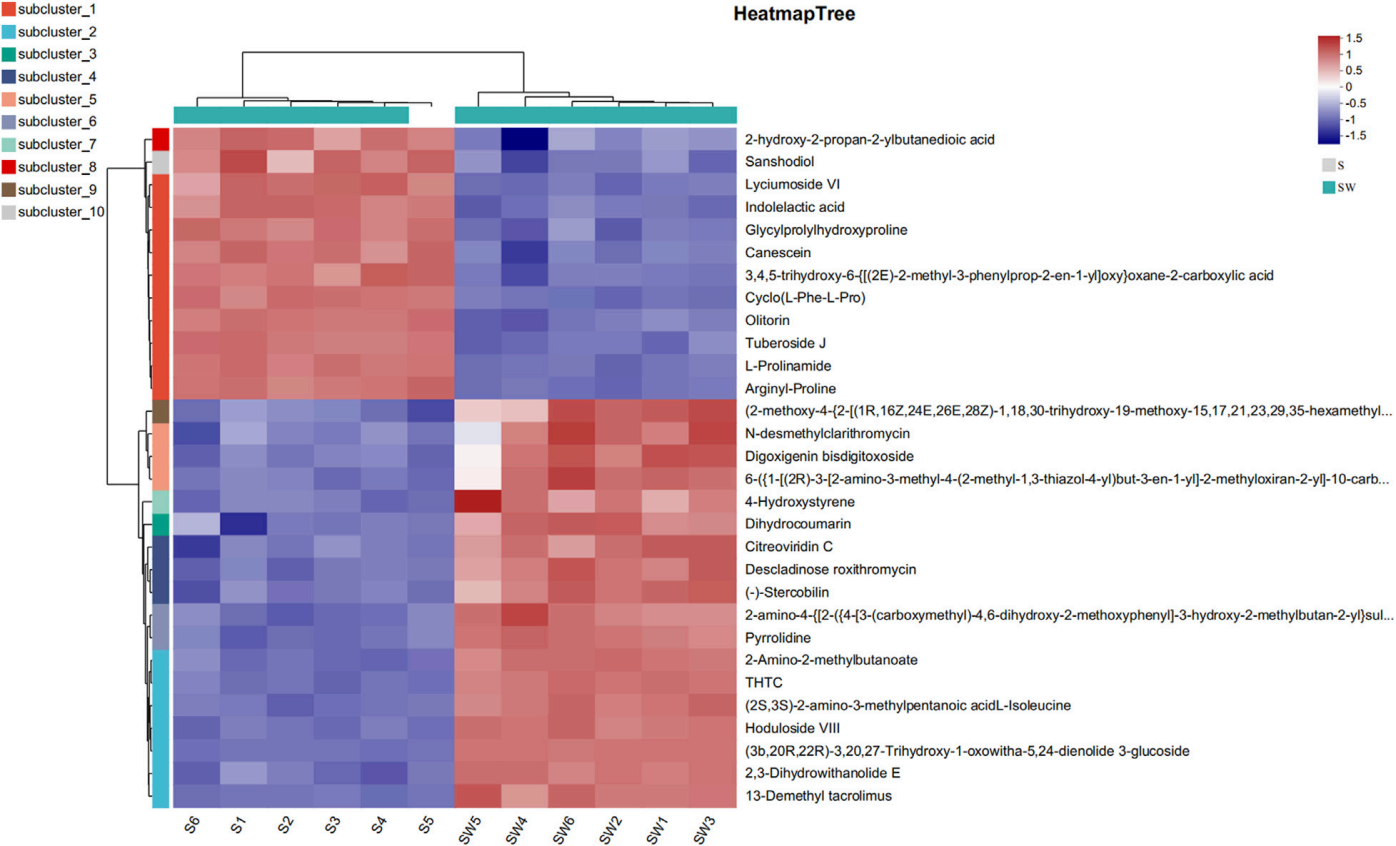
Heatmap analysis of top 30 differential metabolites.

Among the 81 upregulated metabolites, carboxylic acids and their derivatives were the most abundant, accounting for 18 species. The following were pregnenolone lipids, accounting for 14 species. The others were triterpenoids, sphingolipids, peptides, nitrogen-containing carboxylic acid derivatives, and amides, respectively (Supplementary Table S1). KEGG compound is a collection of small molecules, biopolymers, and other chemicals associated with biological systems. As shown in Figure 6B, the differential metabolites can be classified into four kinds of metabolites, including lipids, nucleic acids, peptides, vitamins, and cofactors. Myristic acid was present in the lipids. There were four nucleic acids, among which were thymidine, deoxyuridine, 5′-CMP, and (2R,3R,4S,5R)-2-(6-aminopurin-9-yl)-5-(hydroxymethyl) oxolane-3,4-diol. There was one peptide, L-tyrosine. There were three vitamins and coenzyme factors, namely, S-adenosylmethionine, pyrroloquinoline quinone, and tetrahydrofolate (Figure 7; Supplementary Table S2).

#### 3.3.3 KEGG enrichment analysis of differential metabolites

Based on the KEGG enrichment analysis, it was found that the different metabolites in the test and control groups were mainly enriched in the following pathways: tryptophan metabolism, one carbon pool by folate, drug metabolism—other enzymes (pyrimidine metabolism), purine metabolism, monobactam biosynthesis, and arginine and proline metabolism pathways. The one carbon pool by folate pathway was the most enriched (Figure 6C).

#### 3.3.4 Heatmap analysis of differential metabolites

Heatmaps constructed based on differential metabolites between the supernatant and autoclaved supernatant of *E. cloacae* showed a clear clustering (Supplementary Figure S2). The autoclaved supernatant showed a higher attracting ability than the *E. cloacae* supernatant, which helped us focus on the upregulated differential metabolite. Figure 6D shows the top 30 differential metabolites, which contain L-prolinamide.

### 3.4 Screening and identification of the attractive active substance of *E. cloacae* against *B. dorsalis*

Based on previous studies, most of the substances found to have attractive activity are pyrazines, amines, ammonia, alcohols, ketones, acids, and phenols. Three different metabolites, namely, 3-indoleacetic acid, 2-hydroxycinnamic acid, and L-prolinamide, were selected for the attractive ability test. These three substances had similar functional groups to methyl salicylate, ME, and N-(3-methylbutyl) acetamide (Table 4), whose attractive ability to *B. dorsalis* has been confirmed.

**TABLE 4:** CAS ID of the metabolite.

Metabolite	CAS ID	Molecular formula	Similar functional groups (the compound with an attractive effect)
L-Prolinamide	7,531-52-4	C_5_H_10_N_2_O	Amidogen, acylamino
3-Indoleacetic acid	87-51-4	C_10_H_9_NO_2_	Benzene ring, imino
2-Hydroxycinnamic acid	614-60-8	C_9_H_8_O_3_	Benzene ring, hydroxy

**FIGURE 8:**
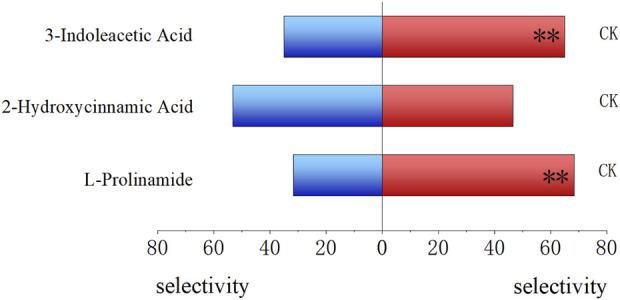
Attraction ability of L-prolinamide, 2-hydroxycinnamic acid, and 3-indoleacetic acid on the larvae of *Bactrocera dorsalis.* Red: control group, where larvae feed on the food normally added with ddH_2_O. Blue: experiment group, where larvae feed on the food added with L-prolinamide, 2-hydroxycinnamic acid, and 3-indoleacetic acid, respectively. *: *P* < 0.05; **: *P* < 0.01.

**FIGURE 9:**
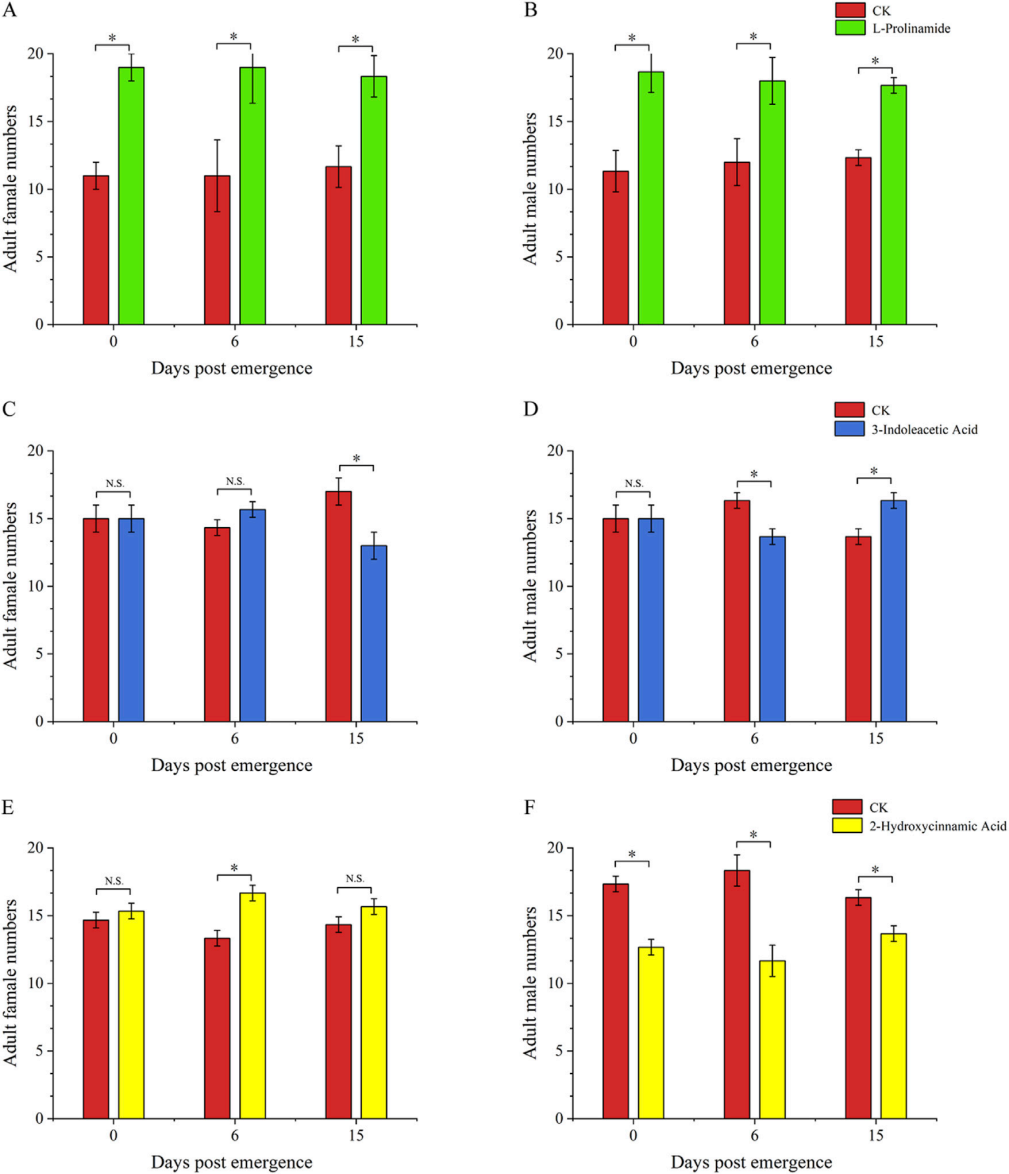
Attraction ability of L-prolinamide, 2-hydroxycinnamic acid, and 3-indoleacetic acid to adult *Bactrocera dorsalis*. Red: control group, where larvae feed on the food normally added with ddH_2_O. Blue: experiment group, where larvae feed on the food normally added with 3-indoleacetic acid. Green: experiment group, where larvae feed on the food normally added with L-prolinamide. Yellow: experiment group, where larvae feed on the food normally added with 2-hydroxycinnamic acid. **(A)** Attraction results of L-Prolinamide to female Bactrocera dorsalis at different ages; **(B)** Attraction results of L-Prolinamide to male Bactrocera dorsalis at different ages; **(C)** Attraction ability of 3-Indoleacetic Acid to female Bactrocera dorsalis at different; **(D)** Attraction ability of 3-Indoleacetic Acid to male Bactrocera dorsalis at different; **(E)** Attraction ability of 2-Hydroxycinnamic acid to female Bactrocera dorsalis at different ages; **(F)** Attraction ability of 2-Hydroxycinnamic acid to male Bactrocera dorsalis at different ages. *: *P* < 0.05. **: *P* < 0.01.

Compared to untreated fresh food, 3-indoleacetic acid-treated food and L-prolinamide-treated food showed a significantly lower attractive ability to the 4-day-old larvae, with an attraction rate of 35.00% (*P* < 0.01) and 31.77% (*P* < 0.01), respectively. The selection rate of 2-hydroxycinnamic acid-treated food (53.33%) and untreated fresh food (46.67%) by 4-day-old larvae had no significant difference (Figure 8). Meanwhile, for the attraction ability of these three substances on female and male adults, L-prolinamide was effective in attracting both newly emerged, 6-day, and 15-day-old males and females, and the number of *B. dorsalis* attracted in the treatment group was significantly higher than that in the control group (Figures 9A, B). 3-Indoleacetic acid showed attractive ability to 15-day-old males (Figures 9C, D), while 2-hydroxycinnamic acid only showed attractive ability to 6-day-old females (Figures 9E, F).

### 3.5 Potential use of L-prolinamide as the synergistic agents of sexual attractants in the field

**FIGURE 10:**
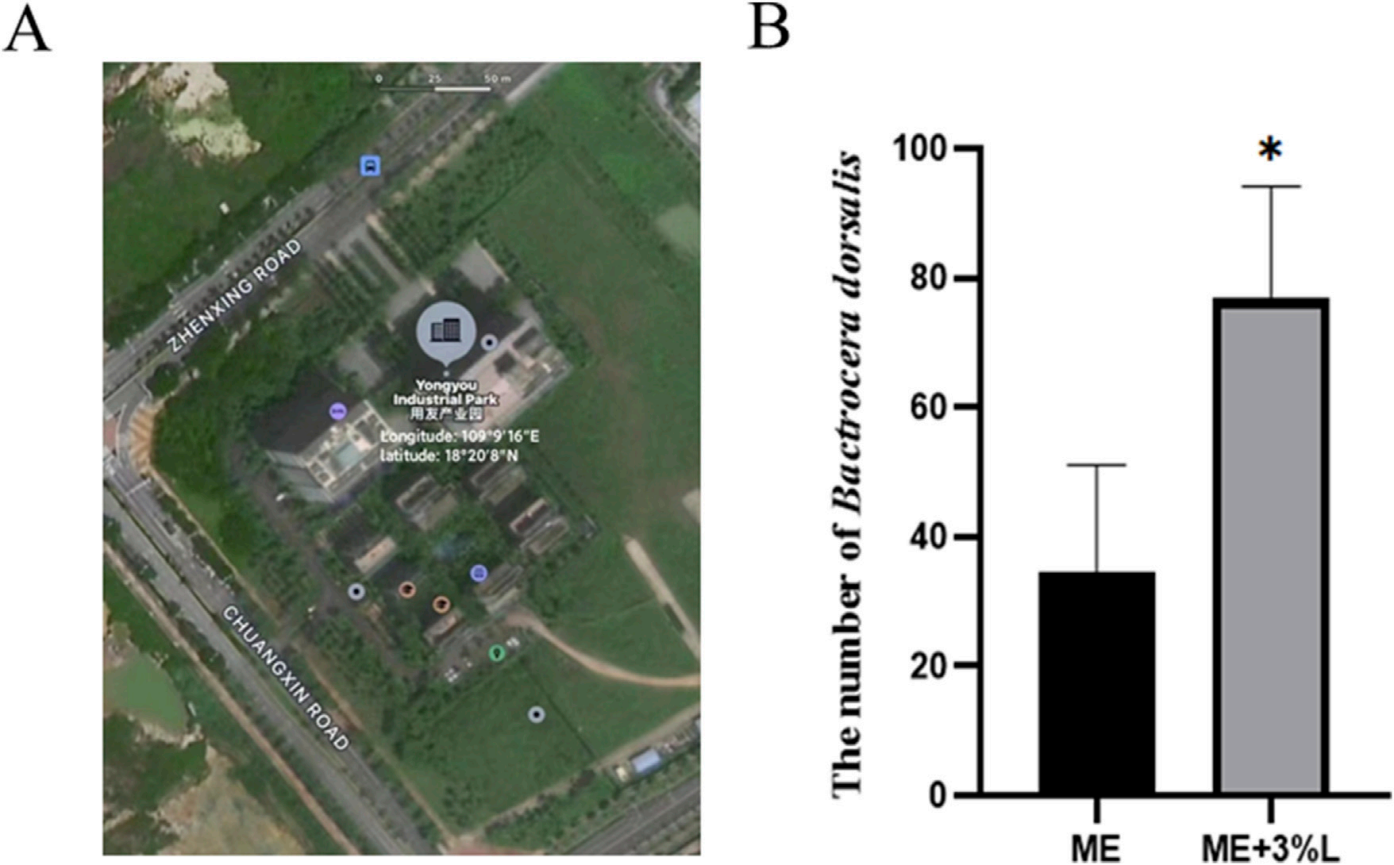
Synergistic effect of L-prolinamide on ME. **(A)** Geographical distribution of the site for field experiment: Yongyou Industrial Park (Longitude:109°9′16″E; latitude: 18°20′8″N); **(B)**: the number of *B. dorsalis* that were trapped using ME and ME mixed with L-prolinamide at a concentration of 3%, respectively. ME, methyl eugenol. ME, control group, only the absorbent cotton, in which 3 mL ME was added, was placed in the small tank for trapping. ME + 3% L: treatment group, besides absorbent cotton with 3 mL ME, the bottom was filled with 100 mL L-prolinamide solution with a final concentration of 3%. The traps were hung on the same branch, and the distance between the control and treatment group was approximately 15 m. N = 16. *: *P* < 0.05.

As shown in Figure 10, the trapping rate of L-prolinamide with a concentration of 3% was significantly higher than that of the control group (n = 16, *P* < 0.05). Therefore, L-prolinamide with 3% concentration had a synergistic effect on ME for the attraction of *B. dorsalis*. A few females were trapped in the treatment group for our pre-experiment, which was constructed in Sanya, Hainan province, in July 2022. However, only 2–7 females were trapped, and it did not display a significant difference.

## 4 Discussion

Wang et al. (2014) identified the attracting ability of gut microbes in adult *B. dorsalis*, and the results showed that Enterobacteraceae, Enterococcaceae, and Bacillaceae have attracting effects on adult *B. dorsalis*, among which *E. cloacae* has a better effect on host fruit fly. In addition, *Bacillus cereus*, *Enterococcus faecalis*, *Citrobacter fumigatus*, *Klebsiella oxytoca*, and *K. pneumoniae* have been reported to be effective in attracting *B. dorsalis* (Jang and Nishijima, 1990; Shi et al., 2012; Shi et al., 2012). *Enterobacter cloacae* also has an attractive effect on other fruit flies. Lauzon *et al.* showed that *E. cloacae*, isolated from the feces of *Rhagoletis pomonella*, has a strong attraction effect on *R. pomonella* (Lauzon et al., 1998). In addition, *E. cloacae* has an attractive effect on *Zeugodacus cucurbitae*, *B. papayae*, and *B. zonata* (Narit and Anuchit, 2011; Reddy et al., 2014). Although the attraction ability of many gut bacteria has been confirmed, the difference of attraction ability among different bacteria species and the specific substances that play a role are unknown. In our research, the results of the larval feeding tendency experiment showed that the 4-day-old larvae were more likely to feed on food that has been fed by themselves or other insects for 4 days, and they also liked the food prepared by the *E. cloacae* solution. Therefore, we speculated that *B. dorsalis* larvae may secrete a substance or pheromone during feeding, which attracted other larvae or themselves to feed. For the attraction to the adult females and males at 0, 6, and 15 days post-emergence, the autoclaved supernatant of *E. cloacae* displayed the highest attraction efficiency. Like our study, it has been shown that *E. cloacae* has a strong attraction effect on 8-day-old and sexually mature South Asian fruit flies. Additionally, research has demonstrated that the autoclaved supernatant is more effective at attracting the flies than the fermentation stock (Luo, 2016).

It has been reported that ammonia, several amines, imines, pyrazines, and acetic acid in the headspace above the filtrate of the bacterial cultures were determined to be attractive to *Anastrepha ludens* (Robacker and Garcia, 1993; Robacker and Flath, 1995). Hereby, we speculated that the fermentation broth of *E. cloacae* can produce a stable secondary metabolite that has an attraction activity on *B. dorsalis* after being autoclaved. Meanwhile, our results showed that there was no significant difference in the attraction effect of the autoclaved supernatant of *E. cloacae* on female and male adults of different ages. This suggests that the active substances responsible for attraction produced by autoclaved *E. cloacae* are not linked to egg laying, sexual maturation, or sex pheromone synthesis. It has been demonstrated that the bacterial medium can provide the fruit flies with carbohydrates and, in turn, can affect the attractiveness of bacteria to fruit flies (Reddy et al., 2014). Meanwhile, the protein-fed or mated fruit flies respond similarly to associated bacterial odors, regardless of the presence or absence of the host fruit (Narit and Anuchit, 2011). Therefore, the attractive substances produced by *E. cloacae* may be some kind of protein or amino acid needed for the growth and development of *B. dorsalis* rather than substances related to sexual maturation and egg production. Therefore, when screening metabolites, combined with previous studies, we guessed that the attractant is the amine-containing substance. Studies of such substances provide a potential use of them as attractants or attractants synergist in the field, which can avoid the disadvantage that some attractants used now only attract males.

Related studies have indicated that most of the substances that have an attraction effect on *B. dorsalis* are substances containing ammonia, pyrazine, amines, alcohols, and phenols. Furthermore, sulfides, ketones, and acids have been reported to be able to attract some fruit fly’s adults (Hadapad et al., 2016). The bacterial volatile components contained in *Anastrepha ludens* also showed significant attraction and were confirmed to contain mainly ammonia, amines, pyrazines, and acids (Robacker and Flath, 1995; Lee et al., 1995; DeMilo et al., 1996; Robacker and Bartelt, 1997; Robacker et al., 1998). Meanwhile, a related study has suggested that the reason some bacteria can have an attraction effect on fruit flies may be due to the production of ammonia by bacterial metabolisms. Meanwhile, amide, as a derivative of ammonia, may have an attraction effect because adult *B. dorsalis* feeds on it as a nitrogen source. Our results were consistent with these reports and findings. Among the three substances we chose, none of them showed attractive ability on larvae, and only L-prolinamide, a kind of amide, showed significantly higher attractive ability on the adult females and males, regardless the development stage.

Although previous studies have shown that most of the substances with an attractive effect on insects are alcohols, phenols, alkanes, and ketones, L-prolinamide has been less studied. Currently, studies have focused on the attraction effect of some other amides, such as *N*-(3-methylbutyl) acetamide and oleamide. It was evident that *N*-(3-methyl-butyl) acetamide induces electroantennagraphical response in different stages and sex combinations in *B. minax* by measuring citrus fruit volatiles and the electroantennographical response of *B. minax* (Li, 2020). A study found that the compound *N*-(3-methylbutyl) acetamide was the main volatile component in the venom of many female vespid wasps. The attraction ability of *N*-(3-methylbutyl) to *Polistes* genera has also been verified by field tests. The acetamide attracted male and female *P. aurifer* and *P. metricus*, as well as male *P. dorsalis* and *P. bellicosus* (Elmquist e al., 2020). The endogenous components of *B. carambolae* males were identified and analyzed for 6-oxo-1-nonanol and *N*-3-methylbutylacetamide, and subsequent studies showed that these endogenous components were able to attract a large number of females when released into the air as visible smoke (Wee and Tan, 2005). It was also found that 1 ng/μL of oleamide had a strong priming effect on the *Curculio chinensis* and identified a regulatory effect of oleamide on its behavior (Yan et al., 2023). In addition to attracting both males and females, L-prolinamide showed a high synergistic effect on the attraction ability of ME by a field test. Its potential use as attractants will be evaluated in the future. This laid a theoretical foundation for the development of new attractants and safe, green, and efficient prevention and control technology of *B. dorsalis*.

Guo et al. (2021) identified the volatile compounds of *Klebsiella oxytoca* and found that 3-methyl-1-butanol only attracted male *Drosophila suzukii*. In contrast, this study has identified a bacterium and its active compound that attract both male and female flies, addressing the current limitation where most attractants only lure males. Additionally, exploring the potential mechanism of *E. cloacae* and L-prolinamide in attracting *B. dorsalis* is critical for pest control. In *Drosophila melanogaster*, the sexually dimorphic central neural pathways associated with the odorant receptor DmelOR67d, which detects cVA, illustrate how olfactory sensory neurons and their projection neurons shape behavior (Datta et al., 2008; Ruta et al., 2010). Zhang et al. (2024) proposed a working model in which ME enhances the attractiveness of leks in attracting females to an arena to control *B. dorsalis* mating behavior, providing a scientific foundation for understanding the mechanisms of the male annihilation technique and future improvements. Given that ME are currently primarily used for managing adult male pests, investigating the potential mechanisms by which *E. cloacae* and L-prolinamide attract female *B. dorsalis* can provide valuable insights for developing pest management strategies. Therefore, further research into these mechanisms could establish a theoretical basis for developing new, environmentally friendly attractants and improving overall control efficacy. In the future, integrating research on *E. cloacae* and its metabolites into current pest management strategies should focus on their broader ecological and pest control impacts. These metabolites demonstrate significant environmental friendliness and protect ecosystem biodiversity compared to traditional chemical attractants. Furthermore, combining these with existing attractants like ME could achieve more effective pest control measures (Cai et al., 2018).

In summary, in this study, the attraction effect of *E. cloacae*, a gut bacterial strain isolated from *B. dorsalis*, and one of its metabolites, L-prolinamide, on host fruit fly was demonstrated. It was confirmed that there was no significant difference between the attraction effect of L-prolinamide on male and female adults, which can compensate for the weakness that most of the current attractants, such as ME, can only attract males, and it can provide a new view for environmental protection and efficient control of *B. dorsalis*. However, among so many differential metabolites, only three substances have been selected to investigate the attraction ability; more differential metabolites should be selected for attraction effect verification, and the comparison among these substances and their synergistic effect of existing attractants still need to be confirmed. Additionally, it is necessary to further investigate the mechanism of the attraction effect of *E. cloacae* and its metabolites on *B. dorsalis* to provide a theoretical basis for the development of novel green control technologies for this notorious pest.

## Data Availability

The original contributions presented in the study are included in the article/supplementary material, further inquiries can be directed to the corresponding author.
