# Supplementary material for: Attractant potential of Enterobacter cloacae and its metabolites to Bactrocera dorsalis (Hendel)
Source: Front Physiol. 2024 Oct 3;15:1465946. doi: 10.3389/fphys.2024.1465946 (PMC11484074; doi:10.3389/fphys.2024.1465946)
Supplement: Supplementary file 2 [file Table1.DOCX]

Supplementary Material

**Supplementary Table 1.** Differential metabolites.

| **Metabolite** | **Molecular Formula** | **VIP** | **Fola change** | **P value** |
| --- | --- | --- | --- | --- |
| Gamma-glutamyl-L-putrescine | C9H19N3O3 | 2.848 | 1.388 | 5.51E-12 |
| Isonicotinamide, 2-propyl- | C9H12N2O | 2.916 | 1.362 | 1.32E-11 |
| Pilocarpic acid | C11H18N2O3 | 2.745 | 1.311 | 3.08E-16 |
| Ganoderic acid Mj | C33H52O6 | 2.704 | 1.264 | 1.89E-09 |
| Uzarigenin 3-[xylosyl-(1->2)-rhamnoside] | C34H52O12 | 2.403 | 1.238 | 0.0002786 |
| 15-Octadecene-9,11,13-triynoic acid | C18H22O2 | 2.254 | 1.227 | 0.0001366 |
| 1,4-Methylimidazoleacetic acid | C6H8N2O2 | 2.739 | 1.221 | 5.61E-16 |
| Convalloside | C35H52O15 | 2.444 | 1.217 | 1.05E-07 |
| Panaxatriol | C30H52O4 | 2.581 | 1.216 | 1.89E-10 |
| L-Prolinamide | C5H10N2O | 2.611 | 1.186 | 5.57E-14 |
| 5-Hydroxyindoleacetylglycine | C12H12N2O4 | 2.050 | 1.156 | 4.45E-12 |
| PE (16:0/0:0) | C21H44NO7P | 2.200 | 1.155 | 1.26E-08 |
| Kanokoside D | C27H44O16 | 2.158 | 1.148 | 7.77E-08 |
| Isoleucyl-Arginine | C12H25N5O3 | 2.123 | 1.146 | 3.89E-12 |
| 5-Hydroxyprimaquine | C15H21N3O2 | 2.091 | 1.140 | 9.87E-12 |
| PE (17:1(9Z)/0:0) | C22H44NO7P | 2.064 | 1.139 | 4.11E-08 |
| Phenylalanyl-Valine | C14H20N2O3 | 1.925 | 1.122 | 9.47E-11 |
| Calligonine | C12H14N2 | 1.871 | 1.121 | 1.41E-08 |
| Lucyoside K | C36H56O9 | 1.963 | 1.120 | 1.38E-09 |
| 1-(2-Furyl) butan-3-one | C8H10O2 | 1.841 | 1.117 | 9.13E-10 |
| Prolyl-Serine | C8H14N2O4 | 1.795 | 1.113 | 2.18E-09 |
| (2b,3b)-Dihydroxy-30-nor-12,20(29)-oleanadiene-28-glucopyranosyloxy-23-oic acid 3-glucuronide | C41H60O17 | 1.893 | 1.102 | 9.75E-12 |
| Imidazolepropionic acid | C6H8N2O2 | 1.809 | 1.100 | 6.50E-13 |
| Hovenidulcioside A2 | C38H58O12 | 1.845 | 1.099 | 3.06E-13 |
| Pterine | C6H5N5O | 1.720 | 1.095 | 1.18E-12 |
| Prolyl-Methionine | C10H18N2O3S | 1.835 | 1.089 | 1.30E-13 |
| Aspergillic acid | C12H20N2O2 | 1.808 | 1.089 | 1.36E-13 |
| Neuromedin B (1-3) | C12H22N4O5 | 1.550 | 1.089 | 2.93E-07 |
| TELMISARTAN | C33H30N4O2 | 1.494 | 1.089 | 0.0001556 |
| 3-trans-p-Coumaroylrotundic acid | C39H54O7 | 1.740 | 1.085 | 3.71E-11 |
| Pyrimidine | C4H4N2 | 1.553 | 1.085 | 8.51E-09 |
| L-2-Amino-5-(methylthio)pentanoic acid | C6H13NO2S | 1.504 | 1.084 | 1.89E-07 |
| S-Adenosylmethionine | C15H23N6O5S+ | 1.464 | 1.084 | 0.0002271 |
| Oxypinnatanine | C10H16N2O6 | 1.574 | 1.083 | 2.01E-08 |
| 2-amino-tetradecanoic acid | C14H29NO2 | 1.410 | 1.083 | 0.0001002 |
| Blumenol C O-[rhamnosyl-(1->6)-glucoside] | C25H42O11 | 1.572 | 1.082 | 1.19E-08 |
| Schidigerasaponin F2 | C39H64O14 | 1.582 | 1.082 | 3.60E-06 |
| Pyrroloquinoline quinone | C14H6N2O8 | 1.336 | 1.078 | 0.003233 |
| Alanyl-Proline | C8H14N2O3 | 1.586 | 1.078 | 2.32E-10 |
| L-Pyridosine | C12H18N2O4 | 1.526 | 1.077 | 5.19E-10 |
| 3-Indoleacetic Acid | C10H9NO2 | 1.472 | 1.076 | 7.68E-10 |
| Cyclo(L-Phe-L-Pro) | C14H16N2O2 | 1.738 | 1.072 | 2.60E-13 |
| N6-Carbamoyl-DL-Lysine | C7H15N3O3 | 1.494 | 1.070 | 3.19E-06 |
| Dihydrolipoamide | C8H17NOS2 | 1.392 | 1.070 | 6.98E-06 |
| Perlolyrine | C16H12N2O2 | 1.395 | 1.068 | 4.37E-08 |
| 4-Acetamido-2-aminobutanoic acid | C6H12N2O3 | 1.277 | 1.066 | 2.64E-05 |
| Physagulin E | C36H50O14 | 1.469 | 1.065 | 9.99E-08 |
| N, N-dimethylhistidine | C8H13N3O2 | 1.404 | 1.063 | 1.92E-10 |
| Mabioside D | C42H70O14 | 1.483 | 1.058 | 3.36E-11 |
| 16-iodo-hexadecanoic acid | C16H31IO2 | 1.311 | 1.056 | 5.76E-06 |
| 2-Methylfuran | C5H6O | 1.243 | 1.055 | 8.39E-10 |
| 4-Hydroxybenzaldehyde | C7H6O2 | 1.243 | 1.055 | 1.01E-10 |
| Alanyl-Tryptophan | C14H17N3O3 | 1.271 | 1.055 | 1.42E-07 |
| Madlongiside C | C35H56O10 | 1.350 | 1.054 | 7.98E-08 |
| 2-(hydroxymethyl)-Pyrimidine | C5H6N2O | 1.194 | 1.053 | 1.59E-06 |
| N-Acetyl-2,3-dihydro-1H-pyrrole | C6H9NO | 1.350 | 1.052 | 1.44E-11 |
| 2-Hydroxycinnamic acid | C9H8O3 | 1.022 | 1.052 | 0.007295 |
| Arginyl-Proline | C11H21N5O3 | 1.530 | 1.051 | 2.32E-13 |
| (3b,22a)-12-Oleanene-3,22,24,29-tetrol 3-[arabinosyl-(1->3)-arabinoside] | C40H66O12 | 1.365 | 1.050 | 1.24E-09 |
| Methylisopelletierine | C9H17NO | 1.275 | 1.045 | 2.69E-09 |
| Barbital | C8H12N2O3 | 1.135 | 1.045 | 5.32E-06 |
| N-Acetyl-DL-tryptophan | C13H14N2O3 | 1.109 | 1.043 | 1.01E-05 |
| 11-alpha-O-beta-D-Glucopyranosyl-16alpha-O-methylneoquassin | C29H44O11 | 1.071 | 1.042 | 7.37E-05 |
| Isoleucyl-Tyrosine | C15H22N2O4 | 1.244 | 1.041 | 8.83E-11 |
| (3-Phenylpropionyl) glycine methyl ester | C12H15NO3 | 1.098 | 1.041 | 5.28E-07 |
| 5'-Deoxy-5-fluorocytidine | C9H12FN3O4 | 1.070 | 1.041 | 3.47E-05 |
| Indolylacryloylglycine | C13H12N2O3 | 1.139 | 1.040 | 1.13E-09 |
| Asparaginyl-Arginine | C10H20N6O4 | 1.087 | 1.039 | 0.0001159 |
| Myricatomentoside I | C26H32O10 | 1.121 | 1.039 | 6.50E-08 |
| 1-Methyladenosine | C11H15N5O4 | 1.096 | 1.039 | 6.35E-07 |
| SR95531 | C15H17N3O3 | 1.161 | 1.039 | 2.84E-08 |
| Ganglioside GD3 (d18:1/16:0) | C68H121N3O29 | 1.106 | 1.037 | 1.82E-06 |
| 6S,9R-Dihydroxy-4,7E-megastigmadien-3-one 9-[apiosyl-(1->6)-glucoside] | C24H38O12 | 1.126 | 1.036 | 8.09E-08 |
| Digitoxigenin 3-[glucosyl-(1->6)-glucosyl-(1->4)-2,6-dideoxyribohexoside] | C41H64O17 | 1.158 | 1.035 | 1.01E-07 |
| Iaa | C10H9NO2 | 1.134 | 1.034 | 5.20E-09 |
| Corchoroside A | C29H42O9 | 1.089 | 1.034 | 4.93E-09 |
| Tuberoside J | C39H64O14 | 1.149 | 1.033 | 8.65E-12 |
| Methionyl-Valine | C10H20N2O3S | 1.035 | 1.031 | 4.13E-07 |
| Cucurbitacin I 2-glucoside | C36H52O12 | 1.040 | 1.031 | 4.68E-08 |
| ALANYL-dl-LEUCINE | C9H18N2O3 | 1.057 | 1.031 | 1.35E-08 |
| Lyciumoside VI | C44H74O20 | 1.043 | 1.026 | 5.97E-10 |

**Supplementary Table 2.** CAS ID of Metabolite

| **Metabolite** | **CAS ID** |
| --- | --- |
| Myristic Acid | 544-63-8 |
| Thymine | 65-71-4 |
| Deoxyuridine | 951-78-0 |
| 5'-CMP | 63-37-6 |
| (2R,3R,4S,5R)-2-(6-aminopurin-9-yl)-5-(hydroxymethyl) oxolane-3,4-diol | - |
| L-tyrosine | 60-18-4 |
| S-Adenosylmethionine | 485-80-3 |
| Pyrroloquinoline quinone | 72909-34-3 |
| Tetrahydrofolic acid | 135-16-0 |


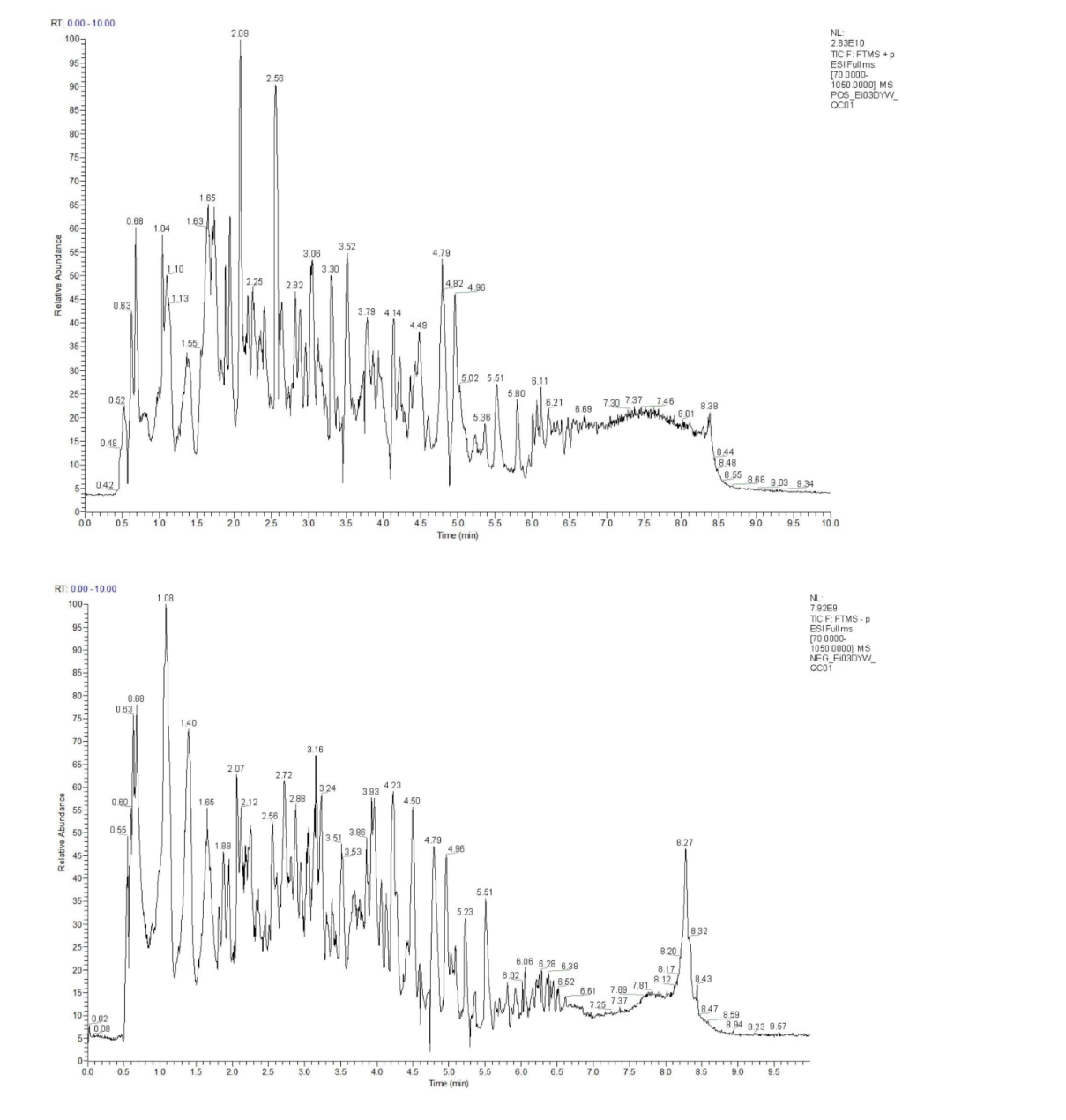


**Supplementary Figure 1.** Total ion flow diagram (+) and diagram (-) of quality control samples.


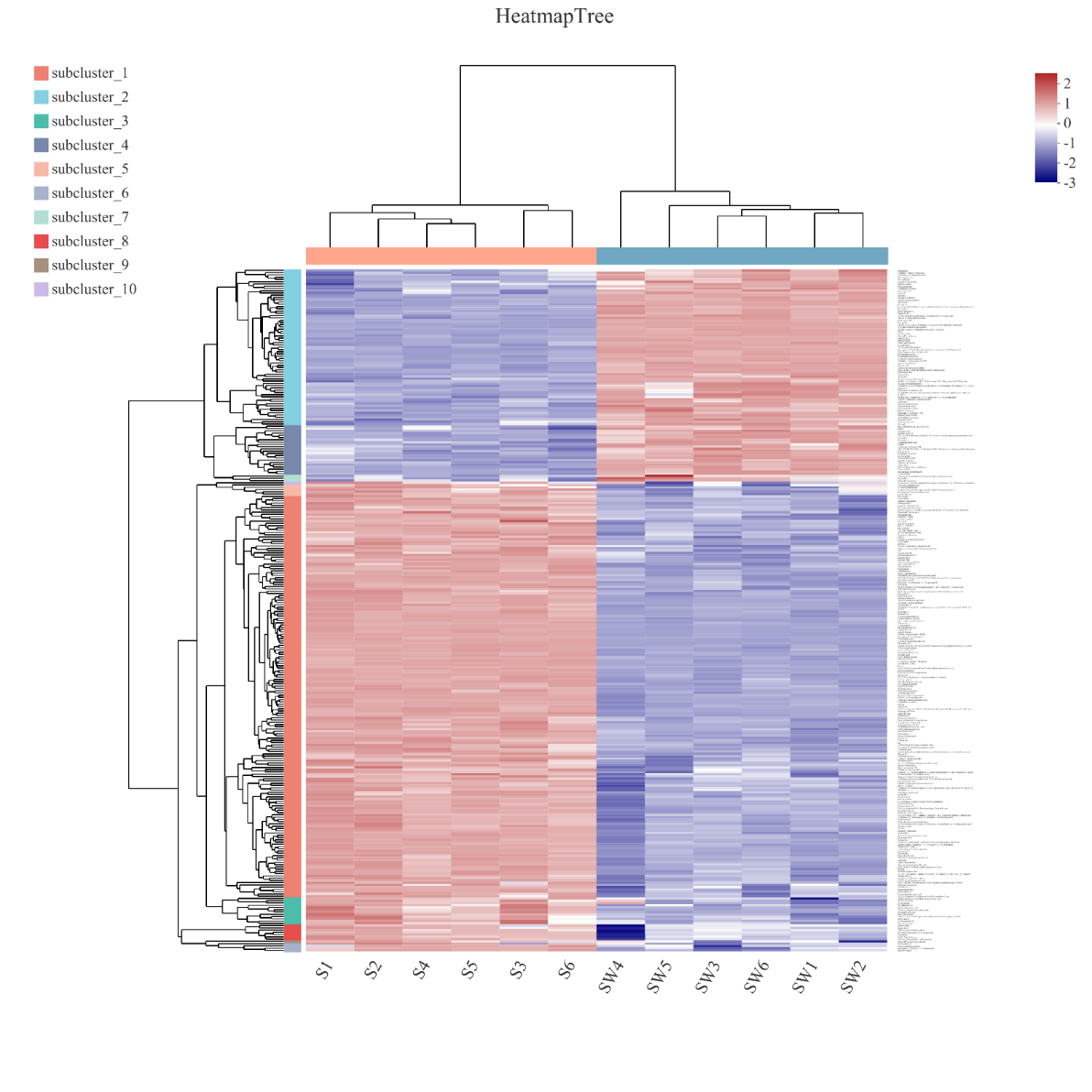


**Supplementary Figure 2.** Cluster analysis results of different samples**.** Each column in the figure represents a sample, each row represents a metabolite, and the color in the figure represents the relative expression amount of the metabolite in this group of samples. On the left is the clustering tree of metabolites, and on the right is the name of metabolites. The closer the branches of two metabolites are, the closer their expression levels are. The upper part is the tree diagram of sample clustering, and the lower part is the name of the sample. The closer the branches of the two samples are, the closer the expression patterns of all metabolites of the two samples are, namely, the closer the change trends of metabolite expression are.
